# Supplementary material for: A fault-intrusion-tolerant system and deadline-aware algorithm for scheduling scientific workflow in the cloud
Source: PeerJ Comput Sci. 2021 Nov 2;7:e747. doi: 10.7717/peerj-cs.747 (PMC8576554; doi:10.7717/peerj-cs.747)
Supplement: Supplemental Information 6 [file peerj-cs-07-747-s006.zip › Myproject1.1/docs/org/cloudbus/cloudsim/power/PowerHostUtilizationHistory.html]

PowerHostUtilizationHistory


JavaScript is disabled on your browser.


- Overview
- Package
- Class
- Use
- Tree
- Deprecated
- Index
- Help

- Prev Class
- Next Class

- Frames
- No Frames

- All Classes

- Summary:
- Nested |
- Field |
- Constr |
- Method

- Detail:
- Field |
- Constr |
- Method


org.cloudbus.cloudsim.power

## Class PowerHostUtilizationHistory

- java.lang.Object
- - org.cloudbus.cloudsim.Host
  - - org.cloudbus.cloudsim.HostDynamicWorkload
    - - org.cloudbus.cloudsim.power.PowerHost
      - - org.cloudbus.cloudsim.power.PowerHostUtilizationHistory

- ---

    

  ```
  public class PowerHostUtilizationHistory
  extends PowerHost
  ```

  The class of a host that stores its CPU utilization history. The history is used by VM allocation
  and selection policies.
  If you are using any algorithms, policies or workload included in the power package please cite
  the following paper:
  Anton Beloglazov, and Rajkumar Buyya, "Optimal Online Deterministic Algorithms and Adaptive
  Heuristics for Energy and Performance Efficient Dynamic Consolidation of Virtual Machines in
  Cloud Data Centers", Concurrency and Computation: Practice and Experience (CCPE), Volume 24,
  Issue 13, Pages: 1397-1420, John Wiley & Sons, Ltd, New York, USA, 2012

  Since:
  :   CloudSim Toolkit 2.0

- - ### Constructor Summary

    Constructors

    Constructor and Description

    `PowerHostUtilizationHistory(int id,
    RamProvisioner ramProvisioner,
    BwProvisioner bwProvisioner,
    long storage,
    java.util.List<? extends Pe> peList,
    VmScheduler vmScheduler,
    PowerModel powerModel)`

    Instantiates a new power host utilization history.
  - ### Method Summary

    Methods

    | Modifier and Type | Method and Description |
    | --- | --- |
    | `protected double[]` | `getUtilizationHistory()` Gets the host utilization history. |

    - ### Methods inherited from class org.cloudbus.cloudsim.power.PowerHost

      `getEnergyLinearInterpolation, getMaxPower, getPower, getPower, getPowerModel, setPowerModel`
    - ### Methods inherited from class org.cloudbus.cloudsim.HostDynamicWorkload

      `addStateHistoryEntry, getCompletedVms, getMaxUtilization, getMaxUtilizationAmongVmsPes, getPreviousUtilizationMips, getPreviousUtilizationOfCpu, getStateHistory, getUtilizationMips, getUtilizationOfBw, getUtilizationOfCpu, getUtilizationOfCpuMips, getUtilizationOfRam, setPreviousUtilizationMips, setUtilizationMips, updateVmsProcessing`
    - ### Methods inherited from class org.cloudbus.cloudsim.Host

      `addMigratingInVm, allocatePesForVm, deallocatePesForVm, getAllocatedMipsForVm, getAvailableMips, getBw, getBwProvisioner, getDatacenter, getId, getMaxAvailableMips, getNumberOfFreePes, getNumberOfPes, getPeList, getRam, getRamProvisioner, getStorage, getTotalAllocatedMipsForVm, getTotalMips, getVm, getVmList, getVmScheduler, getVmsMigratingIn, isFailed, isSuitableForVm, reallocateMigratingInVms, removeMigratingInVm, setBwProvisioner, setDatacenter, setFailed, setFailed, setId, setPeList, setPeStatus, setRamProvisioner, setStorage, setVmScheduler, vmCreate, vmDeallocate, vmDeallocateAll, vmDestroy, vmDestroyAll`
    - ### Methods inherited from class java.lang.Object

      `clone, equals, finalize, getClass, hashCode, notify, notifyAll, toString, wait, wait, wait`

- - ### Constructor Detail


    - #### PowerHostUtilizationHistory

      ```
      public PowerHostUtilizationHistory(int id,
                                 RamProvisioner ramProvisioner,
                                 BwProvisioner bwProvisioner,
                                 long storage,
                                 java.util.List<? extends Pe> peList,
                                 VmScheduler vmScheduler,
                                 PowerModel powerModel)
      ```

      Instantiates a new power host utilization history.

      Parameters:
      :   `id` - the id
      :   `ramProvisioner` - the ram provisioner
      :   `bwProvisioner` - the bw provisioner
      :   `storage` - the storage
      :   `peList` - the pe list
      :   `vmScheduler` - the vm scheduler
      :   `powerModel` - the power model
  - ### Method Detail


    - #### getUtilizationHistory

      ```
      protected double[] getUtilizationHistory()
      ```

      Gets the host utilization history.

      Returns:
      :   the host utilization history


- Overview
- Package
- Class
- Use
- Tree
- Deprecated
- Index
- Help

- Prev Class
- Next Class

- Frames
- No Frames

- All Classes

- Summary:
- Nested |
- Field |
- Constr |
- Method

- Detail:
- Field |
- Constr |
- Method
